# A contemporary review of the management strategies for sickle cell disease related ischaemic and stuttering priapism

**DOI:** 10.1038/s41443-024-01008-z

**Published:** 2024-12-21

**Authors:** Anthony Emmanuel, Ahmed Moussa, Rachel Kesse-Adu, Majed Shabbir

**Affiliations:** 1 Department of Urology, Guy’s & St Thomas’ Hospital, London, UK; 2 Department of Haematology, Guy’s and St Thomas’ Hospital, London, UK; 3 Faculty of Life Sciences & Medicine, King’s College London, London, UK

**Keywords:** Urogenital diseases, Disease prevention

## Abstract

Sickle cell disease is one of the most common autosomal recessive genetic disorders with 23% and over 70% of men with this condition, experiencing episodes of ischaemic priapism and stuttering priapism, respectively, with potentially severe consequences. The effective prevention of sickle cell disease induced ischaemic priapism and stuttering priapism requires a multidisciplinary and multimodal approach. A search of the English literature was performed utilising Pubmed® and Google Scholar to identify publications on contemporary and novel treatment options, with their associated treatment outcomes if available, that are utilised to prevent stuttering priapism episodes and hence a fulminant ischaemic priapism. This narrative review focuses on three main aspects which include firstly, patient education and lifestyle modifications. Secondly, strategies aimed at preventing stuttering priapism episodes with traditional treatments such as alpha-adrenergic agonists and hormone manipulation strategies among others. Finally, we review treatments utilised to treat the underlying sickle cell disease with contemporary options such as hydroxyurea to more novel therapies such as crizanlizumab and voxelotor. The role of potentially curative techniques such as gene therapy and stem cell transplantation are also reviewed and summarised.

## Introduction

Sickle cell disease (SCD) is one of the most common autosomal recessive genetic disorders worldwide. The prevalence of homozygous SCD carriers worldwide is estimated between 6.5–9.2 million in 2021 [1]. It is associated with significant morbidity, mortality, and healthcare-associated costs [1, 2]. The mechanism of sickle cell crises (SCC) involves the polymerisation and sickling of red blood cells which triggers a vaso-occlusive crisis (VOC) that can affect any area in the body including the penis leading to ischaemic priapism (IP) and/or stuttering priapism (SP) [3].

IP results in a painful compartment syndrome of the penis and as time progress irreversible tissue necrosis, fibrosis and lasting functional damage with refractory erectile dysfunction (ED) [4–6]. However, SP typically occurs at night, and are usually self-limiting ischaemic episodes lasting <30–60 mins. Recurrent SP can result in psychological distress, sleep disturbances and over time, lead to tissue damage and hence, ED [4].

In adults, SCD accounts for 23% of all IP cases and this rises to 63% in the paediatric population [7]. Overall, between 72–90% of patients with SCD experience SP, with a mean age of onset of 15 years old and up to 90% of men having their first episode before the age of 20 [8, 9]. Idris et al. [10] found 87% of men with SCD reported a psychological impact of their SP, including fear, anger, frustration, depression, anxiety and embarrassment. Anele and Burnett [11] found that SCD men who experience SP were five times more likely to develop ED, even with episodes lasting less than 2 hours.

**Fig. 1:**
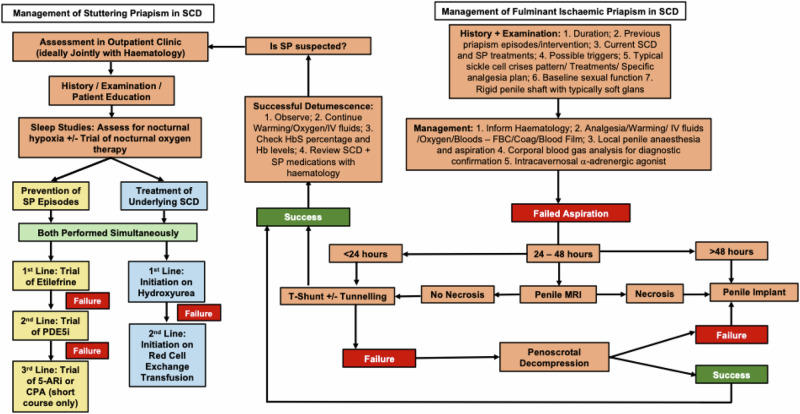
Algorithm for the management of fulminant ischaemic priapism and stuttering priapism in a patient with sickle cell disease (Edited from [4]). SCD sickle cell disease, SP stuttering priapism, IP ischaemic priapism, sickle cell haemaglobin, Coag coagulation Screen, FBC full blood count, PDE5i phosphodiesterase 5 inhibitors, CPA cyproterone acetate, 5-AR 5 alpha reductase.

However, only 5% of men recall ever learning about IP/SP as a potential complication of their SCD [12]. This to an extent may explain why so many present late to emergency services as, Bennett and Mulhall [12] found 92% and 33% of SCD patients present with IP after 12 hours and >24 hours, respectively. Sadly, Idris et al. [10] reported that 49% of surveyed men with SCD never had treatment for their IP. Delayed presentation and treatment are associated with a lower chance of successful detumescence and a greater risk of developing irreversible smooth muscle necrosis and subsequent ED, a devastating complication in a young man [10, 13]. Therefore, prompt management of IP in SCD patients is paramount following recognised guidelines to avoid delay and the potential need for a penile prosthesis (Fig. 1).

Ultimately, the prevention of IP/SP in SCD patients is the key objective [5] and this requires a multi-modal management approach focused on three main areas which include: 1) improved patient and carer education, avoidance of triggers and lifestyle modifications; 2) treatments aimed at preventing SP episodes and 3) treatments aimed at managing the underlying SCD (Fig. 1).

This paper provides a narrative review of the historical and conventional treatments in preventing SP episodes in SCD. In addition, we summarise the novel treatments strategies for SCD which may have a future role in preventing SP as research in this field develops and evaluate potentially curative techniques for SCD such as gene therapy or stem cell transplantation and their current/future clinical utility.

## Methods

A Pubmed® and a Google Scholar library search of the English language literature was performed to identify publications on current and novel treatments of SP in SCD and also of the underlying condition itself from January 1974 to June 2023. Emphasis was placed on articles describing treatment and patient outcomes in the prevention of SP episodes in SCD patients. Hands searching of the grey literature for references of existing literatures reviews was also carried out. Titles, abstracts and full text articles in the English language literature were screened by two individuals (AE and AM) for inclusion in the narrative review. The review was divided into three key areas:

1. Patient education and lifestyle modifications
2. Strategies aimed at preventing SP episodes
3. Treatments aimed at managing the underlying SCD

## Discussion

### Patient education and lifestyle modifications

#### Patient education, medication and lifestyle management

Patient education and improved understanding of the importance of early presentation, when IP episodes last < 2 h, is essential at reducing the risk of long-term damage and progressive ED in SCD [5]. Given the increased risk of developing IP/SP episodes during puberty [9], the education of SCD patients from a younger age is paramount to prevent irreversible damage before adulthood [14]. To highlight this issue a novel joint collaboration between Guy’s Hospital (London), The Sickle Cell Society and Boston Scientific developed an educational video aimed at boys, young men, their carers and also healthcare professionals to increase understanding, encourage early healthcare engagement by breaking the stigma and embarrassment of reporting these events and improve clinician awareness and counselling [15].

Patients who report having an IP or SP episodes require a review of their concurrent medications both prescribed and self-directed with attention to any recreational drugs. Where possible changing to an alternative medication is key, with adequate education and counselling to avoid causative medications/drugs [4].

#### Night-time oxygen levels

Over 40% of adult SCD patients experience sleep disordered breathing, which encompasses conditions like obstructive sleep apnoea leading to momentary night-time oxygen deficiency [16]. This can progress and initiate a SP episode during typical night-time erections. The UK SCD standard of care advise sleep studies for those with SCD displaying signs of disturbed sleep breathing which includes snoring, unwarranted daytime fatigue, early morning headaches and SP [14]. Those with abnormal sleep study results should be referred to a specialist respiratory physician to review and discuss the merits of night-time oxygen supplementation, which can reduce SP episodes in those prone to nocturnal hypoxia [17–19].

### Strategies aimed at preventing stuttering priapism episodes

#### Alpha-adrenergic agonists

Alpha-adrenergic agonists, notably etilefrine, have been employed in treating SCD related SP [4, 5]. Their action lies within the induction of smooth muscle contraction of the penile helicine arteries of the corpus cavernosa, hence facilitating an increase in venous return leading to penile detumescence [20].

A previous prospective study by Okpala et al. [21], highlighted a role for etilefrine in 18 patients with SCD who experienced SP. They found 13/18 (72%) patients had a good clinical response with a reduction in the frequency and severity of SP episodes with no patients developing hypertension or ED. Their regime involved taking etilefrine 25 mg modified release (MR) formulation at night and if SP persisted increased to 50 mg MR at the 2-week period. Blood pressure monitoring during treatment is crucial to detect any episodes of secondary hypertension [21].

A recent cohort study by Johnson et al. [22] had similar findings in 49 patients with SCD who were established on etilefrine therapy, with 34/49 (69%) showing a partial response and 3/49 (6%) a complete response to treatment, respectively. However, due to the short half-life of the only available immediate release etilefrine, 8 patients reported enhanced symptom management by taking a further split dose of 15 mg at 22:00 and 15 mg at 02:00 to reduce early morning SP episodes [22].

#### Phosphodiesterase type 5 inhibitors

Phosphodiesterase type 5 inhibitors (PDE5i) function by facilitating smooth muscle relaxation in the corpus cavernosum and enhancing blood flow to the penis resulting in its effective use as a treatment for ED. Sickle cell mice studies have found aberrant NO signal transduction pathways in SCD, with NO imbalance and a resultant reduction in PDE5 expression contributes to SP development in this condition [23, 24]. This theory led to the apparent paradoxical use of daily PDE5i in men with SCD in an effort to promote an upregulation of the PDE5 enzyme and to restore normal penile vascular homeostasis to prevent SP [24, 25]. In 2006, a feasibility study of PDE5i in IP retrospectively reported on only 4 men with SCD with 2 years follow-up; 3/4 reported only very rare SP episodes while on treatment (tadalafil 10 mg alternate days or daily sildenafil 25 mg) [25].

More recently, a retrospective study reported on using daily 25 mg sildenafil in the morning for between 3–7 months in 24 SP patients of which 46% had SCD (11/24) [26]. They found that treatment reduced emergency department visits, SP duration and frequency across the group. 92% (22/24) reported some improvement in SP outcome while 38% (9/24) reported complete resolution of SP. However, no separate subgroup analysis was performed on the 11 SCD patients in this study to allow targeted analysis on the specific effect of regimented PDE5i administration in this cohort. We have found PDE5i to be only of very limited benefit in SCD related SP in our centre.

#### Hormonal manipulation

##### Cyproterone acetate

Cyproterone acetate (CPA), a steroidal anti-androgen, acts by blocking androgen receptors and causes a centrally mediated reduction in testicular secretion of androgens by suppressing LH [27]. There is scarce evidence for its use and only a single case report by Alshahrani [28] is available in the current literature. He reported that a three-week course of 50 mg CPA daily, subsequently reduced to a once-a-week 50 mg dose helped to prevent further IP episodes for the next two years. In this case the patient had a normal erection with minimal side effect, though adverse effects can occur which include reduced libido, gynaecomastia, a loss of normal functional erections, hot flushes and more rarely cardiovascular disturbances, thromboembolism [28]. Meningioma has also been reported, but the risk is increased with higher cumulative doses [29].

With the potential for serious adverse events related to the side-effect profile of CPA, its use is largely limited to short treatment durations in the well-informed and agreeable patients.

##### 5-α reductase inhibitors

Testosterone within the body is converted to its more potent form dihydrotestosterone (DHT) by the enzyme 5-α reductase (5AR) which are inhibited by finasteride and dutasteride [30, 31]. Both are associated with side-effects such as gynaecomastia, ED, reduced libido and low mood among others [31].

Rachid-Filho et al. [30] used finasteride in 35 SCD patients with SP occurring more than twice a week of at least 40 minutes duration. They used a finasteride reduction treatment regime of 5 mg once-a-day (OD) for 40 days then 3 mg OD for following 40 days then 1 mg OD for the final 40 days and reported a reduction in the frequency of SP episodes from a mean of 22.7 down to 2.1 episodes over 120 days. Similarly, a retrospective study by Baker et al. [31] assessed the effectiveness of dutasteride in preventing SP in 13 patients, of which only 4 had SCD. They found 3/4 (75%) SCD patients compared to 2/9 (22%) of non- SCD required progression to aggressive treatments such as androgen deprivation therapy and penile prosthesis, suggesting dutasteride may be more efficacious for non-SCD patients. However, as the study was significantly underpowered, no strong conclusions could be drawn about the clinical utility of dutasteride in SCD.

##### Ketoconazole

Ketoconazole (KTZ) is an oral anti-fungal medication that has the capability to suppress androgen synthesis in the testes and adrenal gland. It is usually co-administered with 5 mg of prednisone to avert adrenal insufficiency, due to complete blockage of adrenal steroid production which can arise with continuous dosing [32].

The largest study of KTZ use was by Hoeh et al. [32] who reviewed 17 patients with SP, of almost daily occurrence, of which only 5/17 (29%) had an SCD aetiology. The authors used a regime of 2 weeks KTZ 200 mg three times-a-day with 5 mg of prednisolone daily and if there were no SP episodes this was reduced to 200 mg KTZ once a night without prednisolone for 6 months with close liver function test monitoring due to the rare risk of hepatoxitcity (< 1% cases). They found 16/17 (94%) of patients had complete resolution of SP whilst on KTZ and 78% had partial or complete resolution of symptoms after discontinuing KTZ with a mean follow-up of 36 months. The study did not perform a sub-group analysis on the SCD patient cohort. No patients experienced any sexual dysfunction though 50% of patients experienced nausea and vomiting and this poor tolerability of KTZ limits its effective use in clinical practice.

##### Diethylstilbestrol

Diethylstilbestrol (DES) is a synthetic non-steroidal oestrogen with anti-gonadotropic effects exerting negative feedback centrally in the pituitary, suppressing the secretion of LH and FSH and hence a reduction in testosterone production [33]. Similar to other hormonal manipulation preventative strategies there is a dearth of contemporary, high-quality evidence for the clinical utility of DES to prevent SP in SCD. Current evidence for its use was reported by Serjeant et al. [34] in 1985 who conducted a double-blinded, placebo-controlled crossover trial in 11 patients with SCD and SP of at least twice a week. They found DES 5 mg daily was superior to placebo in preventing SP episodes and when all patients crossed over to DES treatment, 8/9 patients (89%) had resolution of their SP attacks with a variable maintenance dose between patients ranging from DES 2.5 mg weekly to alternate days administration to prevent further attacks. However, the low patient numbers and lack of intention-to-treat analysis meant this study lacked sufficient quality for firm conclusions to be drawn.

Similarly, a case report by Gbadoe et al. [35] found in 2 patients with SCD and SP that with a DES regime similar to Serjeant et al. [34], both patients had good initial results but on dose reduction of DES to 1 mg weekly their SP returned. Therefore, they failed to attain a minimum DES threshold dose which allowed SP prevention and maintain normal erections. The side effects associated with DES including gynaecomastia and the increased risk of venous thromboembolism have limited its use in contemporary practice [35].

### Treatments aimed at managing the underlying sickle cell disease

#### Conventional treatments

##### Hydroxyurea

The cornerstone of medical management and usually the first line treatment for SCD remains hydroxyurea (HU), which acts via multiple cellular mechanisms to decrease endothelial adhesion and vasoconstriction leading to a reduction of haemoglobin S (HbS) polymerisation, an increase in fetal haemoglobin (HbF) and reduced red blood sickling [27]. A seminal multi-centre placebo-controlled trial by Charache et al. [36], found HU significantly reduced the median crises rate by 50% but also the number of acute chest syndrome events (ACSE), the need for transfusions, and hospitalisations. Adverse effects of HU include skin discoloration and leg ulcers. However, its negative impact on fertility deters many young men from starting this medication, despite the option of sperm cryopreservation prior to treatment and the reversible nature of its effect on spermatogenesis [37].

Though there are no clinical trials on the efficacy of HU in relation to prevention of SP, HU has been shown, in a small cross-sectional study of only 12 SCD patients, to decrease the frequency of SP episodes in up to 63% of patients with complete cessation of SP in 42% of patients [38]. A variable dose range of HU has been demonstrated to lead to SP resolution in multiple case reports. Saad et al. [39] found between 20 and 35 mg/kg in 5 SCD patients was required for SP resolution, while other groups reported clinical effectiveness with daily doses of 1 g and 1.5 g, respectively [40, 41].

##### Red cell exchange therapy

Blood transfusion in SCD improves the ability to transport oxygen, corrects anaemia and reduces the tendency for VOC and haemolytic episodes [42]. Red cell exchange (RCE) is a process whereby venesection is undertaken simultaneously with blood transfusions aiming to reduce HbS to <30% which results in a significant reduction in the proportion of circulating sickle cells [42]. The stroke prevention (STOP) RCT found that chronic transfusion was effective in reducing stroke risk in children with SCD, and RCE was shown to reduce incidence of pain (40% in the placebo group vs. 27% in the RCE arm) and ACSE (21% placebo arm vs. 6% in the RCE group) [43].

RCE is not the primary treatment method for IP due to the risk neurological complications [44]. However, in a study of 10 patients with acute IP, RCE with a target of Hb ∼10 g/dL and HbS <30% resolved IP in 7/10 patients [42]. By keeping the post-RCE target Hb at ∼10 g/dL, the risk of serious neurological events was mitigated in this study with no patients developing this serious complication, despite earlier reports of such events in studies which did not prevent Hb rises >10 g/dl [42]. However, RCE is best used as an adjunct to treatment of acute IP rather than the primary form of treatment in the acute setting. The reduction in the circulating sickle cells after RCE reduces the risk of recurrent priapism, and treats the underlying factor driving priapism formation. It may form a longer-term preventative strategy for controlling crises in those who cannot accept HU or where HU fails and allows better control of the patient’s overall condition.

#### Potential novel therapies

##### Crizanlizumab

Crizanlizumab is an intravenously administered humanised monoclonal antibody, which binds to P-selectin and blocks its interaction with P-selectin glycoprotein ligand 1. Thus, inhibiting P-selectin initiated adhesion of sickled erythrocytes to the vascular endothelium and a subsequent VOC [45]. The SUSTAIN trial [45], a 12 month-follow-up double blinded, placebo-controlled RCT which reported on 198 patients, compared Crizanlizumab to placebo and found high dose crizanlizumab (5 mg/kg) lowered the rate of SCD crises by 45.3%, with a longer median time to first crises compared to placebo (4.07 vs 1.38 months, *p* = 0.001). In contrast to many other therapeutic options in SCD, crizanlizumab had a low incidence of adverse events [45].

Crizanlizumab has also recently been shown to have a potentially role in reducing SP. Interim results from the open label phase II SPARTAN study [46] included 24 SCD patients aged ≥12 with SP episodes lasting ≥60 minutes over the 14-week preceding the trial. All 24 SCD patients were given crizanlizumab 5 mg/kg over a 26-week period. 70% (17/24) of patients witnessed a 53% reduction in SP episodes relative to their pre-treatment baseline [46], raising hope for this new therapy in managing SCD related SP. It remains to be seen where crizanlizumab fits in to current treatment protocols and regimes for SCD related SP/IP, an area with very few new drugs over the last 20 years.

##### Voxelotor

Voxelotor is another new development for management of SCD. It is an HbS polymerisation inhibitor that reversibly binds to stabilise the oxygenated Hb state, preventing red blood cell sickling [47]. It was approved by the Food and Drug Administration (FDA) for use in SCD patients ≥ 16 years old after the international double-blinded, placebo-controlled phase 3 HOPE RCT in 2019. This trial recruited 274 SCD patients with a Hb between 5.5 g/dL–10.5 g/dL and 1-10 VOC in the last 12 months. They were randomly assigned to one daily oral dose of 1500 mg of voxelotor or 900 mg voxelotor or placebo in 1:1:1 ratio for 72 weeks. It found a significantly higher percentage of Hb response (defined as increase in >1 g/dL from baseline at 24 weeks) in patients on 1500 mg OD voxelotor compared to placebo (51% vs 7%) and a reduction in haemolysis consistent with inhibition of HbS polymerisation and a potential disease modification effect [47]. Unfortunately, despite these promising results no clinical trials have yet been performed assessing the merits of voxelotor as a preventative agent in SCD related SP, and its specific role in treating priapism is yet to be clearly defined.

##### L-Glutamine

L-glutamine is a naturally occurring amino acid within the human body and its exogenous use has been shown to reduce the oxidative stress involved in the pathophysiology of SCD by increasing the proportion of the reduced form of nicotinamide adenine dinucleotides in sickled erythrocytes, therefore resulting in fewer episodes of SCD related pain [48]. A multicentre, double-blinded, phase 3 RCT by Niihara et al. [48] in 230 patients with >2 SCD related pain episodes were randomised 2:1 to L-glutamine (oral twice a day, ~0.3 kg body weight per dose) and placebo over 48 weeks. They found compared to placebo, patients on L-glutamine had a 25% lower cumulative number of pain crises (*p* = 0.005) and fewer ACSE (23.1% vs 8.6%). However, there are no published papers or trials in the available literature that have assessed the potential use of L-glutamine as a preventative agent in SP, and its place in the treatment protocols for priapism are yet to be clarified.

#### Treatments with curative intent

##### Allogeneic haematopoietic stem cell transplantation

Allogeneic hematopoietic stem cell transplantation (HSCT) is the only established cure for SCD. It involves the administration of healthy HSC from ideally a matched human leukoctye antigen (HLA) donor into a patient with a dysfunctional bone marrow [49]. Conditioning prior to HSCT differs according to the intensity of chemotherapy-induced bone marrow suppression [50].

Myeloablative (MA)-HLA matched-sibling HSCT yields the best results in children with possible attenuation/reversal of end organ damage. Outcome data shows overall survival (OS) and disease free-survival (DFS) close to 95% [51]. This forms the basis of the American Society of Haematology’s guideline that this should be the standard of care in children <15 years old with symptomatic SCD [52]. However, in adults, a non-myeloablative (NMA)-HLA matched-sibling HSCT is the preferred approach, due to reduced toxicity and risk of infertility, whilst still retaining a DFS of 87% [53].

However, only <20% of SCD patients have an HLA matched-donor, thus further research into possible alternative donor HSCT sources are being evaluated [54].

##### Gene therapy

Gene therapy is an attractive treatment for SCD, given the condition’s monogenic point mutation. This strategy has the potential for cure but avoids the issues of graft failure and Graft-versus- host disease of HSCT. Two broad approaches exist for minimising the effect of the β^s^ mutation by gene modification termed gene addition and gene editing [55]. Both aim to allow lifelong production of normal Hb or Hb with anti-sickling properties (such as increases in levels of HbF, which is associated with a milder SCD phenotype) [55].

There are ongoing clinical trials into gene therapy in SCD, some of which are aimed at reactivation of HbF such as the CLIMB-SCD-121 Phase 1/2 trial [56]. The results from a single SCD patient with 7 VOC and 5 transfusions per year over two years, was free from these events at 15 months of treatment with HbF levels 43.2% vs. 9.1% at baseline and total Hb 12 g/dL vs 7.2 g/dL at baseline. She did however have over 100 adverse events such as cholelithiasis, neutropenic sepsis which resolved with medical treatments and the study is currently still actively recruiting. Though promising further research in gene therapy is required to optimise long-term safety, its efficacy and portability before its widespread implementation.

## Conclusion

Despite being one of the most common single gene disorders, advances in the management of SCD and its complications have been scarce. Preventative strategies are key in reducing the resultant sequalae of SP events and there should be an emphasis on early education with a multidisciplinary and multi-modal approach ideally in a dedicated joint specialist SCD clinic with haematology and urology.

Multiple conventional treatments exist for prevention of SP in SCD though based on small studies with low quality evidence. The management of SP should be aimed at reducing and preventing episodes, while also managing the underlying condition. To our knowledge there are no current guidelines/algorithms available on how to approach managing SP in a clinical setting. From our experience in our tertiary level specialised SCD priapism clinic, treatment with nightly alpha-adrenergic agents forms an acceptable first line option which is generally well tolerated by patients, with the addition of hormonal therapy in the form of finasteride or CPA for short term use where required. Concurrent management aimed at improving SCD in the form of HU first line, followed by RCE in those unable or unhappy to use this treatment is the mainstay of therapy (Tables 1 and 2). The lack of trials has been an issue in SCD management, but recently well-designed studies has led to FDA approvals of new drugs including crizanlizumab and voxelotor.

**Table 1:** Summary of the treatment strategies for the prevention of stuttering priapism.

Treatment	Focus of therapy	Mechanism	Best evidence	Role in therapy^a^
Alpha -adrenergic Agonists	Prevention of SP episodes	Smooth muscle contraction of the penile helicine arteries of the corpus cavernosa, facilitating an increase in venous return leading to penile detumescence	Retrospective cohort study 49 pts, 69% PR 6% CR [22]	1st line prevention
PDE5i	Prevention of SP episodes	Upregulation of the PDE5 enzyme and to restore normal penile vascular homeostasis	Retrospective cohort study 24 pts, 11 with SCD. 38% CR, but no SCD subgroup analysis [26]	2nd line prevention
5-AR inhibitors	Prevention of SP episodes	Prevents conversion of testosterone to more potent form DHT	Retrospective cohort study 35 SCD pts, SP episode reduction from 22.7 to 2.1 over 120 days [30]	3rd line prevention
CPA	Prevention of SP episodes	Blocks androgen receptors and causes a centrally mediated reduction in testicular secretion of androgens by suppressing LH and hence erections	Single Case Report only – 3 week course of CPA daily then once a week prevented further IP episodes [28]	3rd line prevention (short course only due to SE profile)
KTZ	Prevention of SP episodes	Suppresses androgen synthesis in the testes and adrenal gland	Retrospective study 17 pts; 5 with SCD; 94% CR; 50% had significant SE [32]	Not used due to SE profile
DES	Prevention of SP episodes	Non-steroidal oestrogen with anti-gonadotropic effects exerting negative feedback centrally suppressing LH and FSH and thus testosterone	Double-blinded, crossover trial 11 pts with SCD; 89% CR compared to placebo [34]	Not used due to SE profile
Crizanlizumab	Prevention of SP episodes	Monoclonal antibody, which binds to and inhibits P-selectin initiated adhesion of sickled erythrocytes to the vascular endothelium thus VOC	SPARTAN Study 24 pts with SCD with SP; 70% had PR with 53% reduction in SP episodes [46]	Not currently defined

*SCD* sickle cell disease, *VOC* vaso-occlusive crises, *SP* stuttering priapism, *IP* ischaemic priapism, *CR* complete response, *PR* partial response, *PDE5i* phosphodiesterase 5 inhibitors, *CPA* cyproterone acetate, *5-AR* 5 alpha reductase, *KTZ* ketoconazole, *DES* diethylstilbestrol, *RCE* red cell exchange, *RCT* randomised controlled trial, *SE* side effect, *LH* leutinizing hormone, *FSH* follicle-stimulating hormone.

^a^From our experience in our dedicated SCD Priapism Clinic at a tertiary level referral centre

**Table 2:** Summary of the treatment strategies for the treatment of sickle cell disease.

Treatment	Focus of therapy	Mechanism	Best evidence	Role in therapy^a^
HU	Treatment of SCD	Decreases endothelial adhesion and vasoconstriction leading to a reduction of HbS polymerisation, increase in HbF and reduced red blood sickling	Cross-sectional study 12 SCD patients, PR 63% CR 42% [38]	1st line treatment
RCE	Treatment of SCD	Venesection is undertaken simultaneously with blood transfusions to reduce HbS to <30% which results in a significant reduction in the proportion of circulating sickle cells	Prospective Study 10 pts with SCD and acute IP; IP resolved in 70% [42]	2nd line treatment
Crizanlizumab	Treatment of SCD	Monoclonal antibody, which binds to and inhibits P-selectin initiated adhesion of sickled erythrocytes to the vascular endothelium thus VOC	SUSTAIN RCT 198 pts with SCD; High dose crizanlizumab lowered rate of SCD crises by 45.3% [45]	Not currently defined
Voxelotor	Treatment of SCD	HbS polymerisation inhibitor that reversibly binds to stabilise the oxygenated Hb state, preventing red blood cell sickling	HOPE RCT 274 pts with SCD High Hb response and reduced haemolysis [47]. No trial on prevention of SP currently.	Not currently defined
L-Glutamine	Treatment of SCD	Reduces the oxidative stress increasing the proportion of the reduced form of nicotinamide adenine dinucleotides in sickled erythrocytes, therefore reduction in VOC	Double blinded RCT 230 SCD pts with >VOC episodes. 25% reduction in pain crises [48]. No trials on prevention of SP currently.	Not currently defined

*SCD* sickle cell disease, *VOC* vaso-occlusive crises, *SP* stuttering priapism, *IP* ischaemic priapism, *CR* complete response, *PR* partial response, *HU* hydroxyurea, *RCE* red cell exchange, *RCT* randomised controlled trial, *HbS* sickle cell haemaglobin, *HbF* fetal haemoglobin.

^a^From our experience in our dedicated SCD Priapism Clinic at a tertiary level referral centre

Although, clinically utility in preventing SP has not yet been established, initial results with crizanlizumab have shown some promise. Progress in curative strategies such as HSCT and gene therapy brings new hope to this field, although their efficacy and widespread utility is yet to be defined.

## Data Availability

All data utilised within this review article can be found in detail within each respective published article within the reference list
